# Sleep aspects and subclinical hypothyroidism: a four-year follow-up of the ELSA-Brasil study

**DOI:** 10.20945/2359-4292-2026-0037

**Published:** 2026-04-01

**Authors:** João Ferreira Silva Junior, Aline Silva-Costa, Maria de Jesus Mendes da Fonseca, Isabela Martins Benseñor, Pedro Guatimosim Vidigal, Sandhi Maria Barreto, Rosane Harter Griep, Aline Araújo Nobre

**Affiliations:** 1 Universidade Estadual do Maranhão: Centro de Estudos Superiores de Itapecuru Mirim, São Luís, MA, Brasil; 2 Universidade Federal Fluminense: Instituto de Saúde Coletiva, Rio de Janeiro, RJ, Brasil; 3 Fundação Oswaldo Cruz: Escola Nacional de Saúde Pública Sergio Arouca, Rio de Janeiro, RJ, Brasil; 4 Universidade de São Paulo: Centro de Pesquisa Clínica e Epidemiológica, São Paulo, SP, Brasil; 5 Universidade Federal de Minas Gerais: Faculdade de Medicina, Belo Horizonte, MG, Brasil; 6 Fundação Oswaldo Cruz: Instituto Oswaldo Cruz, Rio de Janeiro, RJ, Brasil; 7 Fundação Oswaldo Cruz: Programa de Computação Científica, Rio de Janeiro, RJ, Brasil

**Keywords:** Insomnia, sleep duration, sleep debt, subclinical hypothyroidism, thyroid disorders

## Abstract

**Objective:**

Previous studies suggest a bidirectional relationship between thyroid dysfunction and sleep disorders. However, prospective evidence regarding the impact of sleep characteristics on subclinical hypothyroidism remains limited. This study aimed to evaluate the association between insomnia symptoms, sleep duration, and sleep debt and the incidence of subclinical hypothyroidism.

**Subjects and methods:**

We conducted a prospective cohort analysis of 7,983 euthyroid participants from the second wave (2012-2014) of the Brazilian Longitudinal Study of Adult Health (ELSA-Brasil) who were not taking thyroid-related or psychiatric medications. Insomnia symptoms (initial, middle, and terminal), sleep duration, sleep debt, sociodemographic characteristics, and health behaviors were assessed via questionnaires. Subclinical hypothyroidism was defined as thyrotropin >4.0 µIU/mL and normal free thyroxine in the third wave (2016-2018). Crude and adjusted log-binomial regression models estimated relative risks (RR) and 95% confidence intervals (95% CI).

**Results:**

The incidence of subclinical hypothyroidism was 6.6% for both sexes. In women, middle insomnia was associated with a 35% reduced risk of subclinical hypothyroidism (RR: 0.65; 95% CI: 0.44-0.92). Among men, sleep debt was linked to a 30% increased incidence (RR: 1.30; 95% CI: 1.01-1.66), and in the continuous model, each additional hour of sleep debt raised the risk by 9% (RR: 1.09; 95% CI: 1.02-1.14).

**Conclusion:**

Of the sleep characteristics assessed, middle insomnia due to nocturnal awakenings appeared to be protective against subclinical hypothyroidism among women, while sleep debt increased the risk among men.

## INTRODUCTION

Subclinical hypothyroidism, a prevalent form of thyroid dysfunction, is characterized by elevated thyrotropin (TSH) levels above the upper reference limit and normal free thyroxine (FT4) levels ^(1,2)^. This condition is commonly observed in clinical practice, with a significant proportion of cases being transient and reversible ^(3)^. Many patients are asymptomatic or exhibit nonspecific symptoms that can be easily mistaken for other conditions, such as obesity, menopause, or depression ^(4,5)^.

Brazilian studies report a prevalence of subclinical hypothyroidism ranging from 5.4% among adults and elderly to 6.5% among adults in the city of São Paulo ^(6,7)^. A 2007 survey in Rio de Janeiro found a prevalence of 12.3% for both clinical and subclinical hypothyroidism in adult women ^(8)^. The condition occurs more frequently in women than men, attributable to hormonal, autoimmune (loss of immunological tolerance), and genetic factors ^(3)^. Female sex hormones influence thyroid function, increasing women’s susceptibility to thyroid diseases ^(9)^. Variations in prevalence are also affected by geographic and demographic factors ^(10-12)^. Advanced age, female sex, and white race/ethnicity are the main risk factors associated with elevated TSH ^(10,11)^. Subclinical hypothyroidism is thus a multifactorial disorder also associated with sleep disturbances ^(13)^.

High-quality sleep is vital for hormonal homeostasis and proper metabolic functioning ^(14,15)^. Endocrine disturbances may directly result from stressors, including poor sleep quality ^(16)^. Sleep disturbances can affect FT4 secretion depending on the severity and duration of the sleep problem, either by activating the hypothalamic-pituitary-adrenal axis or by modulating TSH levels - the primary marker of subclinical hypothyroidism - via negative feedback in the hypothalamic-pituitary-thyroid (HPT) axis ^(17)^.

Several studies over the past decade have shown that acute sleep restriction elevates serum TSH and reduces FT4 and free T3 ^(17-19)^. Conversely, chronic sleep restriction may induce adaptation, resulting in decreased TSH ^(18)^. An experimental study found that sleep deprivation may cause hypothyroxinemia (a reduction in T4), a state that can precede hyperthyrotropinemia (increased TSH) ^(19)^. Regarding insomnia, a cross-sectional study did not find differences in TSH between individuals with and without insomnia ^(20)^. Another population-based cross-sectional study observed that short and long sleep duration did not significantly affect TSH compared to normal sleep duration (7-8 hours); however, long sleep duration increased the odds of subclinical hypothyroidism by 97% ^(13)^.

Given the bidirectional relationship between sleep and the endocrine system ^(15,21)^, both clinical and subclinical thyroid dysfunction are associated with sleep abnormalities. Individuals with clinical hyperthyroidism often experience insomnia and short sleep, while those with clinical hypothyroidism exhibit excessive somnolence and prolonged sleep duration ^(22-24)^. Nonetheless, a cohort study of 682 older men found no differences in subjective or objective sleep measures when comparing individuals with subclinical thyroid diseases to euthyroid controls ^(25)^. Although sleep and thyroid hormones are interdependent under normal physiological conditions ^(15)^, health behaviors such as short sleep and sleep debt, which deregulate various hormones, may precede subclinical thyroid disease. Thus, the directionality of the association, in which sleep disturbances precede subclinical hypothyroidism, is also plausible, albeit understudied ^(25,26)^. It is not known thus far whether insomnia symptoms, analyzed prospectively, deregulate TSH levels.

Given the above, insomnia symptoms, sleep debt, and extremes of sleep duration may be risk factors for subclinical hypothyroidism, though few studies have addressed this direction of causality ^(10,13,14,17,20)^. The few existing studies differ in design and sleep-related exposures assessed. Therefore, we investigated the association between insomnia symptoms, sleep duration, and sleep debt, and the incidence of subclinical hypothyroidism in a cohort of Brazilian public employees, stratified by sex.

## SUBJECTS AND METHODS

### Study design

We conducted a prospective cohort analysis using data from the Brazilian Longitudinal Study of Adult Health (ELSA-Brasil), a multicenter study conducted in five universities and one research center across six Brazilian states (Bahia, Espírito Santo, Minas Gerais, Rio Grande do Sul, São Paulo, and Rio de Janeiro). ELSA-Brasil’s primary objective is to investigate the incidence and progression of chronic noncommunicable diseases, particularly diabetes and cardiovascular disease. Participants are active and retired public employees aged 35-74 at baseline (more information, see https://www.elsabrasil.org).

### Context and participants

The baseline assessment occurred between 2008 and 2010 and included 15,105 participants. The second wave (2012-2014) comprised 14,014 participants, and the third wave (2016-2018) included 12,636 participants of both sexes. This analysis utilized data from waves 2 and 3, as sleep data were unavailable at baseline. We only considered euthyroid individuals from wave 2 who were not using medications known to alter thyroid hormones (amiodarone, biotin, hydrocortisone, prednisolone, dexamethasone, phenobarbital, phenytoin, divalproex sodium, valproic acid, primidone, haloperidol, heparin, furosemide, carbidopa, levodopa, lithium, metoclopramide, carbamazepine, oxcarbazepine, or rifampicin) ^(27,28)^, antithyroid drugs (methimazole or propylthiouracil), or undergoing thyroid hormone replacement therapy (levothyroxine, L-thyroxine, or liothyronine) ^(28)^.

**Figure 1:**
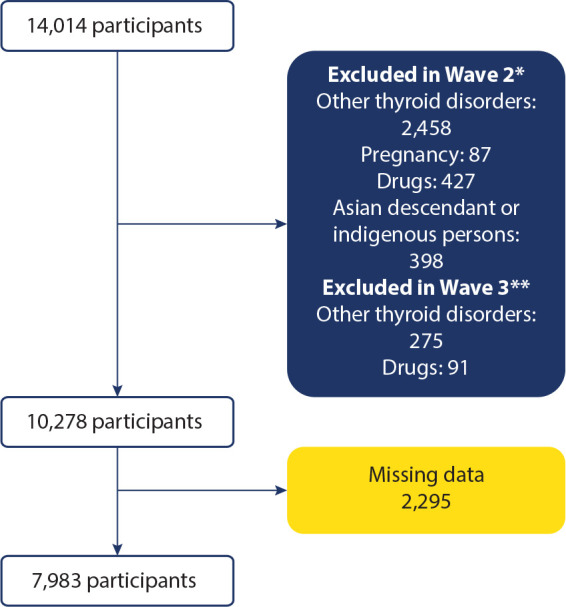
Flowchart of selected participants.

Participants using psychotropic medications, including antidepressants (tricyclics, selective serotonin reuptake inhibitors, serotonin-noradrenaline reuptake inhibitors, monoamine oxidase inhibitors, and atypical antidepressants), mood stabilizers, antipsychotics, tranquilizers and sedatives or hypnotics, as well as medications for attention-deficit/hyperactivity disorder (e.g., methylphenidate, lisdexamfetamine, and amphetamine salts) or autism spectrum disorder, were excluded. Individuals of Asian or indigenous descent were also excluded due to their small numbers (2.56% and 0.95%, respectively). Pregnant women and individuals with missing data for key variables were also excluded. Data from wave 3 included euthyroid individuals or those with subclinical hypothyroidism who were not using the aforementioned drugs and did not have missing data. After all exclusions, the final sample comprised 7,983 participants followed for four years (**Figure 1**).

*In Wave 2, individuals with clinical or subclinical hyperthyroidism, clinical or subclinical hypothyroidism, or central hyper-/hypothyroidism (hypopituitarism) were excluded. Individuals using medications known to affect thyroid function (e.g., amiodarone, biotin, hydrocortisone, prednisolone, dexamethasone, phenobarbital, phenytoin, divalproex sodium, valproic acid, primidone, haloperidol, heparin, furosemide, carbidopa, levodopa, lithium, metoclopramide, carbamazepine, oxcarbazepine, rifampicin, methimazole, propylthiouracil, liothyronine, or levothyroxine) were also excluded. Further exclusions were applied to individuals taking anxiolytics (e.g., bromazepam, clonazepam, diazepam, or lorazepam), antidepressants (e.g., SSRIs, SNRIs, or tricyclics), antipsychotics, or Z-drugs, as well as to pregnant individuals and those of Asian or Indigenous descent. **In Wave 3, individuals with subclinical hypothyroidism were not excluded; all other exclusion criteria remained unchanged.

### Subclinical hypothyroidism

Incident subclinical hypothyroidism was defined as normal thyroid function during the second wave of ELSA-Brasil (0.4-4.0 µIU/mL for TSH and 0.93-1.7 ng/dL for FT4), followed by TSH >4.0 µIU/mL and normal FT4 during the third wave. TSH and FT4 were measured in centrifuged serum samples collected after 12 hours of overnight fasting, using a third-generation immunoenzymatic assay (Roche Diagnostics, Germany) ^(27)^.

### Insomnia symptoms, sleep duration, and sleep debt

Sleep variables were measured using a questionnaire in the second wave of ELSA-Brasil. Insomnia was assessed with questions addressing initial (sleep-onset) insomnia (“*In the last 30 nights, have you had difficulty falling asleep?*”), middle insomnia (“*In the last 30 nights, have you woken up and had difficulty falling asleep again?*”), and terminal insomnia (“*In the last 30 nights, have you woken up before you wanted and been unable to fall asleep again?*”) ^(29)^. Responses (never, rarely, sometimes, almost always, and always) were categorized as “yes” if “always” or “almost always”, and “no” otherwise. Each question was analyzed separately, as well as a composite variable indicating any insomnia symptom.

Sleep duration was assessed with the question: “*How many hours on average do you sleep on a normal night?*” Responses were numeric and participants were classified in three groups short (≤6 hours), normal (>6 and ≤8 hours), and long (>8 hours) sleep duration ^(30)^. Sleep debt was measured as the difference between desired and actual hours of sleep, based on the question “*How many hours would you like to sleep to feel refreshed?*” ^(31)^. A difference was then categorized as “no” when it was ≤1 hour and “yes” when it was >1 hour. Sleep debt was also analyzed as a continuous variable, with negative values resulting from the difference between the questions set to zero.

### Confounders and effect modification

Adjustment variables were also measured with the questionnaire in the second wave of ELSA-Brasil and selected based on the literature for cross-sectional studies and included age, race/ethnicity (white, brown, or black), education (higher, secondary, or primary), smoking (non-smokers, former, or current), alcohol consumption (non-drinkers, former, or current), physical activity (intense, moderate, or light), coffee consumption (never, once a day, 2-3 times a day, and >3 times a day), and menopause (for females).

Alcohol consumption was assessed using the questions “*Do you currently consume alcohol?*” and “*Have you ever consumed alcohol?*”. Participants who reported current consumption were classified as “current drinkers”, those who reported past consumption were classified as “former drinkers”, and those who answered “no” to both questions were classified as “non-drinkers”.

Physical activity was measured using the International Physical Activity Questionnaire, to estimate the weekly time spent in light, moderate, and intense physical activities. Coffee consumption was using a question from the food consumption questionnaire, and answers were categorized as never/almost never, ≤1/day, 1-3/day, and >3/day.

Menopause was measured with closed questions on women’s menstrual and defined as a cessation for >6 months or self-reported natural menopause. Given the higher prevalence of sleep problems and thyroid diseases in women, analyses were stratified by sex, which was considered an effect-modifying variable ^(32)^.

### Statistical analysis

Descriptive analysis used absolute and relative frequencies (incidence) for categorical variables and means and standard deviations for the quantitative variables, sleep deprivation and age. Incidence was calculated as the number of new cases among exposed individuals over the total exposed for each variable. Bivariate analysis employed Pearson’s chi-squared test for categorical and Student’s *t*-test for continuous variables.

Crude and adjusted log-binomial regression models were used to evaluate whether insomnia symptoms (overall and by type), sleep duration, and sleep debt increased the risk of subclinical hypothyroidism. Log-binomial regression was used to estimate the relative risks (RRs) and 95% confidence intervals (CIs). Model 1 adjusted for age, race/ethnicity, and education; Model 2 was adjusted for age, race/ethnicity, education, smoking, alcohol consumption, physical activity, and coffee consumption. The models for female sex were also adjusted for menopause. All analyses were conducted separately by sex using R software version 4.2.1 (R Foundation for Statistical Computing, Austria).

### Ethics

This study was approved by the Ethics Committee at all six research centers. All participants provided written informed consent.

## RESULTS

Over the four-year follow-up, the incidence of subclinical hypothyroidism was 6.6% in both men and women. Among women, the incidence was 5.4% among those with any insomnia symptoms, 5.6% for initial insomnia, 4.6% for middle insomnia, 6.1% for terminal insomnia, 6.6% for short sleep, 9.5% for long sleep duration, and 6.2% for sleep debt. Among men, the respective incidences were 6.1% (any insomnia),6.4% (initial insomnia), 6.0% (middle insomnia), 6.6% (terminal insomnia), 6.4% (short sleep), 9.3% (long sleep), and 7.1% (sleep debt) (**Table 1**).

**Table 1:** Incidence of subclinical hypothyroidism according to subjective characteristics of sleep, sociodemographic conditions, and health behaviors, stratified by sex. Brazilian Longitudinal Study of Adult Health (ELSA-Brasil, 2012-2018)

Variables		Women (n = 4112; 51.5%)		Men (n = 3871; 48.5%)	
		Euthyr.	Shypo.	Euthyr.	Shypo.
**n (%)**		3,839 (93.4)	273 (6.6)	3,615 (93.4)	256 (6.6)
**Continuous variables**					
Age^*^	Mean (SD)	54.6 (8.50)	55.8 (8.55)	54.3 (8.80)	56.6 (9.23)
Sleep duration	Mean (SD)	6.49 (1.39)	6.55 (1.46)	6.41 (1.25)	6.54 (1.32)
Sleep debt	Mean (SD)	1.65 (1.69)	1.64 (2.24)	1.26 (1.55)	1.42 (1.87)
**Categorical variables**					
Insomnia symptoms	No	2,813 (92.9)	214 (7.1)	2,950 (93.3)	213 (6.7)
	Yes	1,026 (94.6)	59 (5.4)	665 (93.9)	43 (6.1)
Initial insomnia	No	3,217 (93.2)	236 (6.8)	3278 (93.4)	233 (6.6)
	Yes	622 (94.4)	37 (5.6)	337 (93.6)	23 (6.4)
Middle insomnia^†^	No	3,192 (93.0)	242 (7.0)	3,194 (93.3)	229 (6.7)
	Yes	647 (95.4)	31 (4.6)	421 (94.0)	27 (6.0)
Terminal insomnia	No	3,249 (93.3)	235 (6.7)	3,247 (93.4)	230 (6.6)
	Yes	590 (93.9)	38 (6.1)	368 (93.4)	26 (6.6)
Sleep duration (hours)	≤ 6	1,926 (93.4)	135 (6.6)	1,933 (93.6)	133 (6.4)
	> 6 and ≤ 8	1,770 (93.5)	123 (6.5)	1,594 (93.3)	114 (6.7)
	> 8	143 (90.5)	15 (9.5)	88 (90.7)	9 (9.3)
Sleep debt	No	1,793 (92.9)	137 (7.1)	2,120 (93.7)	142 (6.3)
	Yes	2,046 (93.8)	136 (6.2)	1,495 (92.9)	114 (7.1)
Race/Ethnicity^*^	White	1,958 (92.2)	166 (7.8)	1,953 (92.6)	157 (7.4)
	Brown	1,065 (94.7)	60 (5.3)	1,128 (94.1)	71 (5.9)
	Black	816 (94.6)	47 (5.4)	534 (95.0)	28 (5.0)
Level of education	Higher	2,247 (93.6)	153 (6.4)	2,037 (93.7)	137 (6.3)
	Secondary	1,299 (93.3)	94 (6.7)	1,124 (92.7)	88 (7.3)
	Primary	293 (91.8)	26 (8.2)	454 (93.6)	31 (6.4)
Alcohol consumption	Non-drinker	746 (92.1)	64 (7.9)	267 (95.0)	14 (5.0)
	Former drinker	680 (93.5)	47 (6.5)	694 (93.9)	45 (6.1)
	Current drinker	2,413 (93.7)	162 (6.3)	2,654 (93.1)	197 (6.9)
Smoking	Non-smoker	2,480 (93.1)	184 (6.9)	1,945 (92.9)	148 (7.1)
	Former smoker	941 (93.6)	64 (6.4)	1235 (93.6)	84 (6.4)
	Current smoker	418 (94.4)	25 (5.6)	435 (94.8)	24 (5.2)
Physical activity	Intense	218 (94)	14 (6)	408 (93.8)	27 (6.2)
	Moderate	612 (93.6)	42 (6.4)	691 (93.1)	51 (6.9)
	Light	3,009 (93.3)	217 (6.7)	2,516 (93.4)	178 (6.6)
Coffee consumption (daily)	Never	301 (92.6)	24 (7.4)	286 (92.9)	22 (7.1)
	≤1	346 (91.8)	31 (8.2)	432 (94.1)	27 (5.9)
	2-3	2,144 (93.6)	147 (6.4)	1,649 (92.8)	128 (7.2)
	>3	1,048 (93.7)	71 (6.3)	1,248 (94.0)	79 (6.0)
Menopause	No	1,300 (94.1)	81 (5.9)	-	-
	Yes	2,539 (93.0)	192 (7.0)	-	-

Euthyr.: Euthyroid; Shypo.: Subclinical hypothyroidism; SD: standard deviation;

* *p*-value< 0.05 for women and men ^†^*p*-value < 0.05 only in women; Chi-square test for categorical variables and Student t-test for continuous variables.

As for sociodemographic and behavioral factors, higher mean age was observed in incident cases compared to euthyroid individuals in both women (mean = 55.8, SD = 8.5) and men (mean = 56.6, SD = 9.2). Incidence was 7.8% in white women, 5.3% in brown women, and 5.4% in black women; for men, 7.4% (white), 5.9% (brown), and 5.0% (black). No other variables showed statistically significant associations (**Table 1**).

Insomnia symptoms, initial insomnia, terminal insomnia, short and long sleep duration, and sleep debt were not significantly associated with incident subclinical hypothyroidism in women. However, women with middle insomnia showed a 35% lower risk of subclinical hypothyroidism (RR: 0.65, 95% CI: 0.44-0.92) compared to those without middle insomnia (**Table 2**). Among men, none of the insomnia exposures or sleep duration categories were associated with subclinical hypothyroidism; however, sleep debt was positively associated. In the fully adjusted model (Model 2), sleep debt increased the risk of subclinical hypothyroidism by 30% (RR: 1.30, 95% CI: 1.01-1.66). In the continuous sleep debt model, each additional hour of sleep debt was associated with an 8% higher risk (RR: 1.08, 95% CI: 1.01-1.13) in Model 1, and a 9% higher risk (RR: 1.09, 95% CI: 1.02-1.14) in Model 2 (**Table 2**).

**Table 2:** Crude and adjusted associations between insomnia symptoms and subtypes, sleep duration, sleep debt, and subclinical hypothyroidism, stratified by sex. Brazilian Longitudinal Study of Adult Health (ELSA-Brasil, 2012-2018)

Variable	Women^†^ (n = 4112; 51.5%)	Men (n = 3871; 48.5%)
**Insomnia symptoms**	**RR (95%CI)**	**RR (95%CI)**
Crude model	0.77 (0.58-1.01)	0.90 (0.65-1.22)
Model 1^*^	0.76 (0.57-1.00)	0.89 (0.64-1.20)
Model 2^*^	0.77 (0.57-1.01)	0.91 (0.65-1.24)
**Initial insomnia**		
Crude model	0.82 (0.58-1.13)	0.96 (0.62-1.42)
Model 1^*^	0.82 (0.57-1.13)	0.98 (0.63-1.45)
Model 2^*^	0.82 (0.58-1.14)	1.01 (0.65-1.49)
**Middle insomnia**		
Crude model	**0.65 (0.44-0.92)**	0.90 (0.60-1.30)
Model 1^*^	**0.65 (0.44-0.92)**	0.86 (0.57-1.24)
Model 2^*^	**0.65 (0.44-0.92)**	0.89 (0.59-1.28)
**Terminal insomnia**		
Crude model	0.90 (0.63-1.23)	1.00 (0.66-1.44)
Model 1^*^	0.90 (0.63-1.24)	0.99 (0.65-1.43)
Model 2^*^	0.90 (0.63-1.24)	1.02 (0.67-1.48)
**Short sleep duration**		
Crude model	1.01 (0.80-1.28)	0.96 (0.76-1.23)
Model 1^*^	1.04 (0.82-1.32)	1.00 (0.78-1.27)
Model 2^*^	1.04 (0.82-1.32)	1.01 (0.79-1.29)
**Long sleep duration**		
Crude model	1.46 (0.84-2.35)	1.39 (0.67-2.49)
Model 1^*^	1.43 (0.82-2.31)	1.37 (0.66-2.45)
Model 2^*^	1.39 (0.79-2.27)	1.34 (0.65-2.42)
**Sleep debt**		
Crude model	0.88 (0.70-1.10)	1.13 (0.89-1.43)
Model 1^*^	0.94 (0.74-1.19)	1.26 (0.99-1.61)
Model 2^*^	0.95 (0.75-1.20)	**1.30 (1.01-1.66)**
**Continuous sleep debt**		
Crude model	1.00 (0.93-1.07)	1.05 (0.98-1.12)
Model 1^*^	1.02 (0.95-1.09)	**1.08 (1.01-1.13)**
Model 2^*^	1.02 (0.95-1.09)	**1.09 (1.02-1.14)**

Notes:

* Model 1 - adjusted for age, race/ethnicity, and level of education; Model 2 - model 1 + alcohol consumption, smoking, physical activity, and coffee consumption;

† Model 2 also adjusted for menopause.

## DISCUSSION

After four years of follow-up, women with middle insomnia exhibited a lower risk of developing subclinical hypothyroidism, whereas sleep debt in men was associated with increased risk. Regarding insomnia and its subtypes, the observed protective effect of middle insomnia among women stood out. Generally, the literature does not support a protective role of insomnia against subclinical hypothyroidism. A recent cross-sectional study reported that individuals with sleep difficulties demonstrated a higher prevalence of hypothyroidism (odds ratio [OR] = 1.38, 95% CI: 1.14-1.68), including the subclinical form, compared to euthyroid individuals ^(33)^. A systematic review corroborates these findings, noting a positive relationship between poor sleep quality and hypothalamic-pituitary-thyroid axis dysfunction, although with some study heterogeneity ^(34)^. Similarly, Mendelian randomization analyses have found no causal association between genetic susceptibility to insomnia and the risk of hypothyroidism ^(35)^, making it biologically unlikely that fragmented sleep would protect thyroid function.

Furthermore, it cannot be ruled out that the finding reflects a chance effect or statistical fluctuations, especially as other insomnia subtypes did not show similar associations. Therefore, caution is warranted in interpreting results and replicating with objective sleep assessments and in other populations is necessary. Notably, an experimental crossover study showed that six weeks of sleep restriction to approximately 6.0 hours per night significantly reduced circulating TSH in women, but not men, with the most pronounced reduction in premenopausal women. Sleep restriction also modestly reduced FT4, with the largest effect size in postmenopausal women, suggesting that insufficient sleep disrupts the HPT axis more in women, possibly due to a more a pronounced proinflammatory response ^(36)^.

Neither initial nor terminal insomnia was significantly associated with subclinical hypothyroidism, although their effect sizes also suggested a protective relationship. Initial and terminal insomnia may have less impact on thyroid hormones because they cause less sleep fragmentation compared to middle insomnia ^(36)^. The scarcity of studies assessing insomnia subtypes hampers a comprehensive interpretation. Still, a cross-sectional study on the relationship between sleep quality (assessed with the Pittsburgh Sleep Quality Index) and TSH levels showed that individuals with poor sleep quality and who took longer to fall asleep had higher odds (OR: 2.39, 95% CI: 1.44-3.98) of subclinical hypothyroidism ^(14)^. These results differ from our findings, as the comparisons are limited since the authors did not stratify by sex and used different measurement tools ^(14)^.

We found no statistically significant association between combined insomnia symptoms and subclinical hypothyroidism. To our knowledge, no comparable longitudinal studies exist. A cross-sectional study on hormone levels in the HPA and HPT axes in individuals with normal thyroid function and insomnia and in healthy controls failed to find a statistically significant difference in TSH levels (2.6 and 2.5 mIU/L, respectively) ^(20)^.

In the current study, insomnia symptoms (jointly or separately) were not associated significantly with subclinical hypothyroidism in men, possibly because they are less susceptible to sympathetic hyperexcitability from sleep fragmentation and thus experience fewer endocrine changes than women ^(37)^.

As for sleep debt, the results suggest an increased risk of subclinical hypothyroidism among men; nevertheless, this association should be interpreted cautiously given the borderline statistical significance of the results. Sleep debt is a stressor with documented effects on well-being and the endocrine system ^(38)^. A recent review showed that a single night of total sleep deprivation can increase TSH levels by up to 200% ^(39)^, whereas partial sleep deprivation produces more modest changes ^(39)^. Chronic partial sleep deprivation affects thyroid hormones which may cause a negative feedback response and decrease TSH level ^(39)^. A study of 32 healthy individuals (16 women) who were sleep deprived for one night noted higher TSH and FT4 levels in both sexes ^(18)^. Another interventional study in 118 individuals showed increased TSH and decreased FT4 after three consecutive days of partial sleep deprivation (4 hours/day) with normalization after four days of recovery sleep ^(17)^. These studies suggest that the duration of deprivation appears to alter FT4 levels differently.

Short or long sleep duration were not associated with subclinical hypothyroidism in either sex. Individual sleep needs vary, and sleep requirements often decline with age. Adults aged ≥40 appear resilient to shorter sleep durations, generally without hormonal disruption. Unlike our findings, a longitudinal and cross-sectional study using the Pittsburgh Sleep Quality Index found that short (<7 hours) and very short (<5 hours) sleep durations were linked to higher TSH levels ^(14)^. Conversely, another cross-sectional study found no higher odds of subclinical hypothyroidism among individuals sleeping <7 hours compared to subjects with normal sleep duration (7-8 hours) although long sleep (>8 hours) increased the odds of subclinical hypothyroidism by 91% (OR: 1.91; 95% CI: 1.03-3.53) ^(13)^.

Importantly, there are clear sex differences in sleep characteristics, as captured by both subjective scales or polysomnography and electroencephalography ^(40)^. Men and women interpret the quality of their sleep differently. For instance, women report more sleep problems and worse quality of sleep, presenting a higher risk of developing insomnia and thyroid disease than men ^(27,37,38)^. Despite women being more likely to complain of insomnia and men reporting short sleep duration more often ^(37,38)^, there is still no definitive explanation for why middle insomnia is only associated with subclinical hypothyroidism in women.

The strengths of this study include the large sample size, measurement of thyroid hormones at two time points using third-generation kits, which are more sensitive for detecting very low hormone levels, and the prospective cohort design, allowed for inferences regarding the exposure’s effect on outcomes among individuals without baseline subclinical hypothyroidism. In addition, the availability of cohort data on medication use facilitated the exclusion of individuals with clinical thyroid diseases, as well as users of drugs that alter thyroid function or psychiatric medication. Despite these strengths, some limitations should be noted, particularly the use of subjective measures for sleep characteristics. Subjective sleep measures are highly susceptible to recall bias and misclassification. Nevertheless, subjective assessment remains the most common approach for measuring sleep characteristics in large cohorts, due to the high cost of objective measures, which are considered the gold standard. Furthermore, among ELSA-Brasil participants, the test-retest reliability of insomnia symptoms and self-reported sleep duration questions demonstrated substantial agreement ^(30)^. Lastly, the ELSA-Brasil study population has higher levels of education and income than Brazil’s general population, as well as greater access to health services. For our study, this may limit only the generalizability of the findings.

Although the prospective design and control of potential confounders strengthen the robustness of the analysis, the inverse direction of the association suggests the possibility of residual bias or unmeasured factors, such as sleep apnea, which may have influenced the observed results. Future research should further investigate the physiological mechanisms underlying the sex-specific differences observed, particularly considering menopausal status as a potential effect modifier in the associations between sleep characteristics and thyroid function. Moreover, incorporating mechanistic biomarkers, such as serum cortisol, proand anti-inflammatory cytokines, and other markers of the HPT axis, could help elucidate the biological pathways linking sleep and hormonal regulation.

Middle insomnia appears to protect women from subclinical hypothyroidism, a finding that warrants further evaluation in future studies. In contrast, sleep debt seems to increase the risk of subclinical hypothyroidism, especially in men. Our study holds significant clinical relevance for public health. Most individuals with subclinical hypothyroidism progress to clinical hypothyroidism within four to five years. Given the uncertainty of signs and symptoms, all risk factors should be identified, mainly due to the increased cardiovascular risk.

## Data Availability

data from the ELSA-Brasil Study is subject to restricted access.
